# Benznidazole Use among Patients with Chronic Chagas' Cardiomyopathy in an Endemic Region of Brazil

**DOI:** 10.1371/journal.pone.0165950

**Published:** 2016-11-17

**Authors:** Ariela Mota Ferreira, Ester Cerdeira Sabino, Lea Campos de Oliveira, Cláudia Di Lorenzo Oliveira, Clareci Silva Cardoso, Antônio Luiz Pinho Ribeiro, Desirée Sant’Ana Haikal

**Affiliations:** 1 Graduate Program in Health Sciences, State University of Montes Claros (Universidade Estadual de Montes Claros), Montes Claros, Minas Gerais, Brazil; 2 Institute of the University of São Paulo, University of São Paulo (Universidade de São Paulo), São Paulo, São Paulo, Brazil; 3 Medical Investigation Laboratories-03 Clinics Hospital, Medical School, University of São Paulo (Universidade de São Paulo), São Paulo, São Paulo, Brazil; 4 Federal University of São João del-Rey, Research Group in Epidemiology and New Technologies in Health-campus CCO, São João del-Rei, Brazil; 5 Department of Internal Medicine, Federal University of Minas Gerais (Universidade Federal de Minas Gerais), Belo Horizonte, Minas Gerais, Brazil; Yeshiva University Albert Einstein College of Medicine, UNITED STATES

## Abstract

Chagas disease (CD) is a neglected tropical disease that affects individuals in almost every country in Latin America. There are two available drugs with antiparasitic profiles; however, only benznidazole (BZN) has been approved for commercialization in Brazil. The usefulness of prescribing BZN for patients with chronic Chagas cardiomyopathy (CCC) is controversial. There are no studies in the literature describing the extent of BZN use at this stage or the profile of patients using this drug. The present study aimed to determine the prevalence and factors associated with previous BZN use among individuals with CCC. This cross-sectional study was conducted with 1,812 individuals with CCC from 21 Brazilian cities endemic for CD. The dependent variable was "prior use of BZN" (no vs. yes). The independent variables were grouped into socioeconomic, lifestyle and medical history aspects. Binary logistic regression (α ≥ 0.05) was used. Among the evaluated individuals, 27.2% reported previous use of BZN. The likelihood of prior use of BZN was higher among younger individuals (OR = 2.7), individuals with a higher education (OR = 2.7), individuals with a lower monthly per capita income (OR = 1.3), individuals who practiced physical exercise (OR = 1.5), individuals who had prior knowledge of the CD diagnosis (OR = 2.5), individuals without hypertension (OR = 1.3) and individuals with a longer time to the CD diagnosis (OR = 6.1). The present study revealed a small proportion of therapeutic BZN use among Brazilian CCC patients. This finding suggests a late diagnosis and undertreatment of the disease. BZN use was higher among individuals with better clinical and demographic conditions but with a lower income and a longer time to the CD diagnosis. Knowledge of the BZN usage profile may help reduce the current state of neglect of this disease and pave the way for future studies.

## Introduction

Chagas disease (CD) is transmitted by *Trypanosoma cruzi* (*T*. *cruzi*) and is a neglected tropical disease that affects 10–12,000,000 individuals in almost every country in Latin America [1]. CD is endemic in rural areas due to economic distortions and social inequalities [2,3]. In Brazil, approximately 2,500,000 individuals are infected with *T*. *cruzi*; therefore, proper medical care must be arranged, particularly for patients living in remote areas. Chronic Chagas’ cardiomyopathy (CCC) occurs in approximately 20 to 40% of infected people [4]. CCC is the most important consequence of CD and is a potentially lethal condition [4,5].

There are only two available drugs with antiparasitic profiles that have been demonstrated to act on *T*. *cruzi*: nifurtimox and benznidazole (BZN) [6]. In Brazil, only BZN has been approved for commercialization, and it can be used by all patients in the acute phase of CD for at least 60 consecutive days [7,8]. In the chronic phase, this drug can be used by patients with the indeterminate form, mild cardiac involvement or the digestive form of the disease [7]. When prescribed, BZN can be obtained free of charge from the Brazilian public health system [9]. Despite reports demonstrating the efficacy of BZN in the acute phase of the infection, clinical results obtained in the chronic phase are more variable, and the occurrence of adverse events is more frequent, particularly in adults [10].

The effectiveness of BZN during the chronic phase of the disease remains controversial, although some benefits of its use during this stage have already been demonstrated [4,11,12]. This treatment does not fully eliminate the parasite, but it reduces the parasite burden, which could benefit the patient as a result of the concomitant attenuation of cardiomyopathy and electrocardiographic changes [4,11]. In contrast, a recent multicenter clinical trial conducted with 2,854 patients with CCC showed a greater negative conversion rate of plasma C-reactive protein (CRP) in the group treated with BZN compared to the placebo group. This result was observed immediately after the treatment and also two and five years after the end of the treatment. However, the rates of conversion to negative PCR results were not associated with a significant reduction in clinical deterioration [12].

Given this controversy, the identification of a CD treatment regimen prescribed for individuals with CCC is relevant [12]. However, there are no studies elucidating the proportion of individuals with CCC using BZN or of the profile of the individuals who underwent this treatment. Therefore, the current study was conducted to test the hypothesis that there is a low proportion of patients with CCC from endemic regions who use BZN. In addition, this study aimed to identify the sociodemographic and clinical characteristics and lifestyle that contribute to BZN use.

## Methods

### Ethical approval

Ethical approval was obtained from the relevant ethics committee (Research Ethics Committee of the Faculty of Medicine of the University of São Paulo—Protocol number: 042/2012). All of the subjects agreed to participate in this study and signed the informed consent form prior to beginning the study.

### Study design

The present research is the baseline of a cohort study that will have a 24-month follow-up; this study is called SaMi-Trop (Study of Biomarkers for Neglected Tropical Diseases of São Paulo/Minas Gerais—Pesquisa em Biomarcadores em Doenças Negligenciadas Tropicais de São Paulo/Minas Gerais) and is a multicenter study involving four Brazilian public universities.

### Study population and recruitment

The present study was conducted in 21 municipalities that were selected because of their high prevalence of CD. These municipalities belong to two mesoregions of the Brazilian state of Minas Gerais that are CD-endemic areas: the northern mesoregion of the state of Minas Gerais and the mesoregion of Vale do Jequitinhonha. These places historically present low socioeconomic and cultural indicators. The Human Development Index (HDI) (0.54 to 0.70, 2010) and the average monthly per capita income (U$275.56, in 2010) of the municipalities included in this study are below the national indexes (0.77 and U$ 466.98, respectively) [13].

The results of electrocardiogram (ECG) tests were used to select the patients for the study. These ECG tests were performed by healthcare professionals who were working in the selected municipalities between 2011 and 2012. Subsequently, these tests were assessed by cardiologists who work in a teleconsulting program that provides healthcare services from a distance (Telehealth Program—Programa Telessaúde), developed by the Telecare Network of Minas Gerais (Rede de Teleassistência de Minas Gerais—RTMG). This program aims to expand the outcomes of the primary healthcare system and to promote its integration with the entire Brazilian healthcare network. It belongs to the public Brazilian healthcare network [14]. After the assessments of the tests, only the individuals over 18 years of age and presenting cardiac abnormalities compatible with CCC were considered eligible. The presence of cardiac abnormalities compatible with CCC included the following abnormalities on the ECG index [15]: possible old myocardial infarction (major Q wave abnormalities or minor Q wave abnormalities with ST segment or T wave abnormalities), complete intraventricular block (right, left or unspecified), frequent supraventricular or ventricular premature beats, major isolated ST segment or T wave abnormalities, atrial fibrillation or flutter, supraventricular tachycardia or other major arrhythmias, major atrioventricular conduction abnormalities or pacemaker use, major QT prolongation (QT index >115%), and left or right ventricular hypertrophy.

A total of 4,689 eligible individuals were identified through the Telehealth Program. Of this total, 2,532 patients were excluded because of inconsistencies in their records, losses, death or refusal to participate in the study. Thus, the baseline of the study consisted of 2,157 individuals. The sample size estimated to observe the main outcomes in this cohort was approximately 2,000 individuals. Compared to the eligible group, the participants had a higher percentage of women (67.9% vs. 59.9%, p<0.01) and younger ages (58.2 vs. 60.7 years, p<0.01).

**Fig 1:**
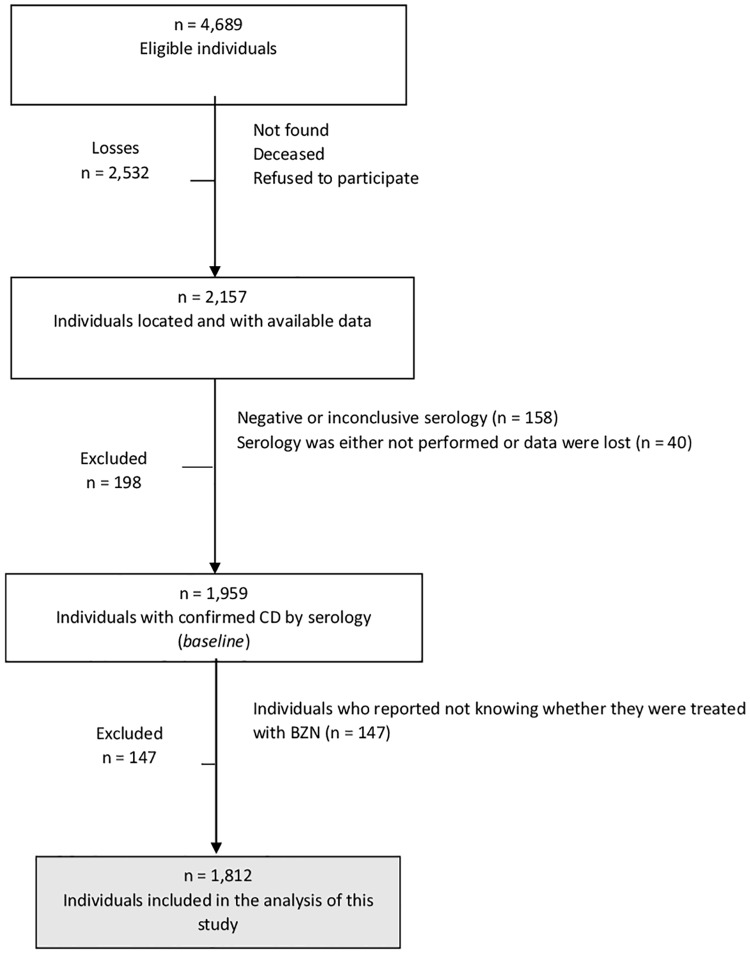
Flowchart of Eligible, Lost and Excluded CD Patients.

All baseline patients were tested for the presence of anti-*T*. *cruzi* antibodies using a chemiluminescent microparticle immunoassay with the Architect Abbott (sensitivity = 99% and specificity = 99.5%). Negative results were reassessed, and immuno-negative results were confirmed by two additional chemiluminescent immunoassays using different antigens (Chagatest v 4.0 Wiener, sensitivity = 100% and specificity = 98.3% and Chagas III GrupoBios Diasorin, sensitivity = 100% and specificity = 100%). Patients with confirmed negative results were excluded from the study. So, only patients who tested positive for the protozoan parasite *T*. *cruzi* were included (n = 1,959). However, 1,812 patients provided information about BZN use and could be included in the present study (Fig 1).

### Data collection

Data were collected between June 2013 and July 2014. The CCC patients were recruited by professionals from the primary healthcare system in each municipality, who scheduled the day and time for the interview and clinical evaluation. The patients’ interviews, peripheral blood collection and ECG tests were performed at the primary healthcare system units. The interview was designed to obtain information regarding demographics, lifestyle, physical exercise, aspects of quality of life, medical history and the CD treatment regimen.

### Statistical analysis

Initially, a descriptive analysis was performed with all of the variables. Simple frequencies (n) and relative frequencies (%) were estimated for each category of variables, and the means and standard deviations were estimated for the variables age and per capita income/month, which were originally numeric variables. Next, a multivariate analysis was performed using a binary logistic regression.

The dependent variable was BZN use, which was obtained from the following question during the interview: “Have you ever taken a drug called BENZNIDAZOLE or ROCHAGAN^®^?” (trade name). The response was obtained directly from the self-report of the participants who responded “No” (for those who have never taken this drug) or “Yes” (for those who reported having taken this drug). A total of 147 individuals who were unable to answer whether they had taken this medication were excluded from the study (Fig 1).

The independent variables were grouped into sociodemographic characteristics, lifestyle and medical history. The variables associated with gender, age, education level, self-declared skin color and per capita income/month were included in the sociodemographic characteristics. Further, per capita income/month was divided into “more than half of the minimum wage” (U$174.87) and “less than half of the minimum wage”, considering the value of the minimum wage in the country at the time of data collection (minimum wage at the time of the data collection: U$349.75–1U$ ≅ R$ 2.07 in December 2012).

Among the lifestyle variables, alcohol consumption, smoking and physical exercise were investigated. Alcohol consumption was assessed with the following question: “How many times have you consumed alcohol in the past thirty days?” The possible responses were “I did not consume it”, “I have consumed it less than once a week” and “I have consumed it more than once a week”. The answers to this question were divided and regrouped into two categories: “No” (no alcohol consumption/alcohol consumption less than once a week) vs. “Yes” (alcohol consumption more than once a week). Smoking was assessed by the question: “Which of the following best describes your cigarette smoking habits?” The possible responses were “I've never smoked”, “I smoked, but now I do not smoke anymore,” or “I currently smoke.” Physical exercise was assessed with the following question: “Physical exercise is a form of physical activity that is planned, structured, and repetitive with professional monitoring, such as swimming, weight training, martial arts or others. Do you practice any physical exercise?” The possible responses were “Yes” or “No”. Thus, the variables smoking (smokers vs. former smoker vs. never smoked) and physical exercise (no vs. yes) remained as they were collected.

The medical history variables were investigated with the patient’s responses addressing prior knowledge of the CD diagnosis, the time since diagnosis (self-reported), the presence of family members diagnosed with CD, the presence of diabetes mellitus and hypertension and the self-perception of health. The time since diagnosis (self-reported) was assessed by the question: “How long ago were you diagnosed with Chagas disease?” The responses were categorized as “less than 5 years” vs. “5 to 10 years” vs. “more than 10 years”. The self- perception of health was assessed by the question “How do you rate your health condition”, using the Likert scale with five answer options that were later divided into negative self-perception (poor/very poor) and positive self-perception (excellent/good/fair).

A bivariate analysis was conducted (Pearson’s chi-square test), and the variables with a p-value ≤ 0.20 were included in the multivariate binary logistic regression model. Only variables with a p-value ≤ 0.05 remained in the final model. The percentage of variance explained by the model was calculated using the adjusted coefficient of determination (pseudo-R^2^). The analyses were conducted using the statistical software Predictive Analytics SoftWare (PASW/SPSS)^®^ version 18.0 for Windows^®^.

## Results

Among the 1,812 patients with CCC, 493 (27.2%) reported the previous use of BZN. Of these, only 325 (65.9%) reported the use of this drug for at least 60 consecutive days. The mean age of the patients was 58.2 (± 12.6) years, and the mean per capita income/month was U$ 161.62 (± 168.39), with 90% of patients earning up to U$ 338.16 per capita/month. Among the 324 (17.9%) individuals diagnosed with CD in the past five years, 21 (1.15%) were diagnosed in the past year. Of these, only 2 individuals reported the use of BZN. The distribution of the patients selected in this study according to sociodemographic characteristics, lifestyle and medical history is shown in Table 1.

**Table 1:** Descriptive and Bivariate Analysis of the Sociodemographic Characteristics, Lifestyle, and Medical History Variables and Their Association with Benznidazole (BZN) Use in Patients with Chronic Chagas’ Cardiomyopathy (CCC) (n = 1,812).

*Characteristics*	*Descriptive*	*Bivariate*		
		Previous use of BZN		
***Sociodemographic***	**n (%)**	**No n (%)**	**Yes n (%)**	**p-value**^π^
Gender				
Female	1,230 (67.9%)	892 (72.5%)	338 (27.5%)	0.705
Male	582 (32.1%)	427 (73.4%)	155 (26.6%)	
Age*				
60 or more years	824 (45.6%)	675 (81.9%)	149 (18.1%)	**<0.001**
40–59 years	859 (47.5%)	561 (65.3%)	298 (34.7%)	
39 or less years	124 (6,9%)	78 (62.9%)	46 (37.1%)	
Self-reported skin color*				
White	391 (21.6%)	292 (74.7%)	99 (25.3%)	0.327
Black/Yellow/Brown/Indigenous	1,413 (78%)	1,020 (72.2%)	393 (27.8%)	
Education level (years of schooling)*				
None	576 (31.8%)	482 (83.7%)	94 (16.3%)	**<0.001**
1–5 years	821 (45.3%)	577 (70.3%)	244 (29.7%)	
6–9 years	312 (17.2%)	199 (63.8%)	113 (36.2%)	
10 or more years	96 (5.3%)	55 (57.3%)	41 (42.7%)	
Marital status*				
Widowed or separated	396 (21.9%)	314 (79.3%)	82 (20.7%)	**0.004**
Single	170 (9.4%)	121 (71.2%)	49 (28.8%)	
Married or stable union	1,157 (63.9%)	819 (70.8%)	338 (29.2%)	
*Per capita* income/month				
More than U$174.87	630 (34.8%)	500 (79.4%)	130 (20.6%)	**<0.001**
Less than U$174.87	1,177 (65%)	814 (69.2%)	363 (30.8%)	
***Lifestyle***				
Alcohol consumption*				
No	1,673 (92.3%)	1,227 (73.3%)	446 (26.7%)	**0.092**
Yes	125 (6.9%)	83 (66.4%)	42 (33.6%)	
Smoking*				
Smoker	124 (6.8%)	97 (78.9%)	27 (21.8%)	0.323
Former smoker	465 (25.7%)	342 (73.5%)	123 (26.5%)	
Never smoked	1,212 (66.9%)	874 (72.1%)	338 (27.9%)	
Physical exercise*				
No	1,393 (76.9%)	1,039 (74.6%)	354 (25.4%)	**0.005**
Yes	407 (22.5%)	275 (67.6%)	132 (32.4%)	
***Medical History***				
Prior knowledge of the CD diagnosis*				
No	60 (3.3%)	53 (88.3%)	7 (11.7%)	**0.006**
Yes	1,737 (95.9%)	1,253 (72.1%)	484 (27.9%)	
Time to diagnosis of CD*				
Less than 5 years	315 (18.3%)	284 (90.1%)	31 (9.9)	**<0.001**
5 to 10 years	345 (20.0%)	252 (73.1%)	93 (26.9%)	
More than 10 years	1,062 (61.7%)	714 (67.2%)	348 (32.8%)	
Family members with CD diagnosis*				
No	365 (20.1%)	273 (74.8%)	92 (25.2%)	**0.001**
Yes	1,295 (71.5%)	919 (71%)	376 (29%)	
Diabetes*				
No	1,645 (90.8%)	133 (79.6%)	34 (20.4%)	**0.037**
Yes	167 (9.2%)	1,186 (72.1%)	459 (27.9%)	
Hypertension				
Yes	1,130 (62.4%)	851 (75.3%)	279 (24.7%)	**0.002**
No	68 (37.6%)	468 (68.6%)	214 (31.4%)	
Health self-perception*				
Negative	242 (13.4%)	184 (76%)	58 (24%)	0.228
Positive	1,550 (85.5%)	1,121 (72.3%)	429 (27.7%)	

* Variation of the n = 1,812 because of missing information.

^π^ Pearson’s chi-squared test

Among the 493 subjects who reported the use of BZN, 303 (61.6%) began this treatment within one month after diagnosis, 93 (18.9%) reported initiating BZN use between one month and one year after diagnosis, 80 (16.3%) reported initiating BZN use one year or more after diagnosis, and 17 (3.3%) were unable to recall this information.

In the bivariate analysis, the variables selected to build the initial multiple model (p ≤ 0.2) regarding BZN use were age, education level, marital status, per capita income/month, alcohol consumption, physical exercise, prior knowledge of the CD diagnosis, time to diagnosis, family members with CD diagnosis and hypertension (Table 1).

The adjusted multiple model revealed that younger individuals (OR = 2.5; 95% CI = 1.5–4.2), individuals with a higher education level (OR = 2.5; 95% CI = 1.5–4.2), individuals with a lower per capita income/month (OR = 1.4; 95% CI = 1.1–1.8), individuals who practiced physical exercise (OR = 1.4; 95% CI = 1.1–1.8), individuals who had prior knowledge of the CD diagnosis (OR = 2.5; 95% CI = 1.1–6.3), individuals without hypertension (OR = 1.3; 95% CI = 1.0–1.6) and individuals with a longer time of diagnosis of CD (OR = 6.2; 95% CI = 4.1–9.4) were more likely to have already used BZN, with the latter variable being more strongly associated with the outcome. This model was able to explain 17% of the variability associated with the previous use of BZN (Table 2).

**Table 2:** Multiple Logistic Regression Model Adjusted for the Variables Associated with Benznidazole (BZN) Use in Patients with Chronic Chagas’ Cardiomyopathy (CCC).

	Previous use of BZN	
Variables	OR [95% CI]	p-value
Age		
60 or more years	1	
40–59 years	2.1 [1.6–2.7]	**<0.001**
39 or less years	2.5 [1.5–4.2]	**<0.001**
Education level (years of schooling)		
None	1	
1–5 years	1.6 [1.2–2.1]	**0.001**
6–9 years	2.1 [1.5–3.0]	**<0.001**
10 or more years	2.5 [1.5–4.2]	**0.001**
*Per capita* income/month		
More than U$174.87	1	
Less than U$174.87	1.4 [1.1–1.8]	**0.028**
Physical exercise		
No	1	
Yes	1.4 [1.1–1.8]	**0.016**
Prior knowledge of the CD diagnosis		
No	1	
Yes	2.5 [1.1–6.3]	**0.048**
Time to diagnosis of CD		
Less than 5 years	1	
5 to 10 years	3.6 [2.3–5.8]	**<0.001**
More than 10 years	6.2 [4.1–9.4]	**<0.001**
Hypertension		
Yes	1	
No	1.3 [1.0–1.6]	**0.050**
*Constant*	-4.60	**<0.001**
Pseudo R^2^ (Nagelkerke)	17%	

## Discussion

The present study demonstrated that barely over a quarter of the patients with CCC had previously used BZN and less than a fifth of them had followed the treatment protocol that recommends a minimum of 60 consecutive days [8,9]. According to our findings, it was also observed that treatment with BZN in the patients with CCC mainly occurred during first year after diagnosis. The previous use of BZN was associated with the variables age, education level, income, physical exercise, prior knowledge of CD diagnosis, time to diagnosis and the absence of hypertension. The proportion of previous use of BZN was higher among individuals with lower income and individuals with a longer time to diagnosis of CD but with better clinical and demographic conditions.

Previous studies reporting the proportion of patients with CCC who were treated with BZN were not identified, preventing both comparisons and the identification of theoretical models that could support the analysis of the current study. The low prevalence of individuals who were treated with BZN may suggest a specific undertreatment of CD, which should have been established during the acute phase of the disease or when there was only mild cardiac involvement [8]. Moreover, one may also suspect a late CD diagnosis, which is detected only after more severe cardiac involvement, as that observed among the 21 patients with CCC who were diagnosed with CD only in the last year.

Whether because of undertreatment or late diagnosis, CD remains a neglected parasitic infectious disease. This situation can be aggravated by the fact that CD occurs primarily in rural areas, where the diagnosis and monitoring of the patients are more difficult because of the lack of healthcare services and trained professionals [16]. Moreover, the divergences observed in the literature about the use of BZN in the chronic phase of CCC [4,11] could contribute to hesitation of the physicians in prescribing this medication for CD treatment. Future studies are needed to clarify these issues.

A interesting observation that deserves attention concerns that among the patients who had used BZN previously, 34% reported that the treatment lasted less than 60 days, which features a frame undertreatment of the disease and, consequently, compromises the effectiveness of treatment. A high dropout rate from BZN treatment was observed in previous studies [12,17–20]. Dropout rates from BZN treatment range from 13% [12] to 34% [18]. Adverse reactions were the main reason for the treatment dropout. A previous longitudinal study revealed that among patients using BZN, only 12.5% had no adverse reactions during treatment, and among those who had an adverse reaction, 25% had to suspend the treatment because of these reactions [19]. The side effects caused by a drug are the most important predictors of its non-adherence [21]. Although dropouts because of adverse effects have been reported in previous studies, failure in prescription, guidance and/or monitoring of these patients during the use of this medication might also being occurring.

Importantly, although *T*. *cruzi* infection does not have a sexual predilection [22], studies show a higher prevalence among women [23,24]. This difference may be related to the use of health services more often by women, even after controlling for restrictions in routine activities due to health reasons [25,26] and the greater availability to participate in scientific studies. This finding is in accordance with the finding that the proportion of women evaluated in this study was greater than the proportion in the identified eligible group (67.9% vs. 59.9%, p<0.01)

In the current study, adult patients were more likely to have used BZN compared to elderly patients. The effectiveness and tolerance of BZN are inversely associated with the age of the patients, with children presenting excellent response rates and a lower incidence of adverse effects, which are relatively rare in this age group [27,28].

Individuals with a longer time of diagnosis of CD are more likely to have already used BZN. In the current study, most patients who were treated with BZN had used it within one year after diagnosis, and more than 60% of these patients were diagnosed for over ten years. According to our findings, only 2 of the 21 patients diagnosed with CD in the last year were treated with BZN. We hypothethize that the rarity of the diagnosis of CD's disease currently, the occurrence of CCC provided between the patients, and the controversy regarding the therapeutic use of BZN for the treatment of these patients may have contributed to the low use of the drug among patients selected in this study [6]. In addition, BZN treatment offered to patients with CD was more prominent in the past decades [29]. CD is considered a typical tropical neglected disease and receives less attention from the media. Moreover, there are few public governmental strategies to combat CD, and these are primarily limited to epidemiological surveillance and the control of blood banks [29].

The lack of health information among patients represents an important barrier to treatment adherence and the successful treatment of neglected diseases. The current study demonstrated that patients with CCC with a higher education level were more likely to adhere to the BZN treatment. Previous studies have demonstrated that higher levels of education are associated with a better understanding of health conditions and the adoption of self-care actions among patients with malaria [30], tuberculosis, HIV/AIDS [31] and individuals with chronic diseases [32]. In the current study, patients with CCC had a low education level (only 5% of the subjects had more than 8 years of schooling). These findings are in agreement with another study conducted in Brazil with CD patients [33]. The current study demonstrated that the chance of BZN use was directly proportional to the increase in the education level of the patients. These data highlight the need to ensure that individuals with lower education levels have access to information about CD; moreover, the data indicate that the State should be absolutely responsible for promoting public education strategies to achieve greater health equity.

The current study revealed that patients with CCC who were aware of the CD diagnosis were more likely to have previously taken BZN. The low prevalence (3%) of patients with CCC who reported no prior knowledge about CD suggests an increase in access to information on the diagnosis, even with the low education level of the patients. Knowledge about the diagnosis of a disease by the patient is essential for the development of self-care skills [30,31,34,35]. Furthermore, the bond between patients and health services is proportional to their discussions about health information and treatments [36,37]. Thus, health services can contribute to the principle of equity by offering a clinical environment that enables the patient to know about CD.

The current study showed that patients with the lowest per capita income had higher adherence to BZN treatment, which was counterintuitive. However, our results are in agreement with a previous study [21] that assessed 2,512 individuals in a representative sample of the German general population and found that nonadherence to medication occurred more often in patients with a higher socioeconomic status [21]. The homogeneity regarding the low income observed in the patients with CCC evaluated in the current study (90% of these patients received a monthly per capita income up to U$338.16) may have contributed to this finding. In addition, an association between a low economic level and CD among patients from rural areas has been reported [38]. This finding can be better explored in future studies that examine the influence of subjective factors related to prescriptions by doctors, which were not assessed in this study.

The data from the current study demonstrated that BZN use was higher among CCC patients who reported not having high blood pressure. High blood pressure can be interpreted by the physician as a poorer health status of the patient [37], which might discourage the prescription of BZN. However, individuals with both CD and high blood pressure could be viewed as at increased risk for the evolution of cardiac involvement [39]. In turn, this condition could encourage the prescription of BZN by healthcare professionals to prevent further heart damage. The cross-sectional design of the current study did not allow us to clarify the cause and effect of this association.

In the current study, patients with CCC who reported performing some type of physical exercise were more likely to adhere to BZN treatment. This finding is in agreement with a previous study that showed a greater adherence to drug therapy prescribed by healthcare professionals among individuals who practice some physical exercise [40].

Importantly, we started with a total of 4,689 patients selected based on the results of ECGs performed in 2011–2012. However, only 2,157 of the patients were located and completed the baseline assessment in 2013–2014. This loss was primarily due to inconsistencies in their records or deaths because the search for these patients took place 2–4 years after the ECG. Although this issue represents a loss inherent to the study design, we must consider the possibility of selection bias. Problems with the quality of the secondary data, such as the absence of information, the illegibility of the medical notes in the medical records, spelling and typographical errors, and incorrect data, have been recognized in the national and international scientific literature [41,42]. We were careful to compare the eligible group with the participant group. We had a higher percentage of women and younger people, as expected. Generally, women are likely to present more complete data records and have a higher life expectancy in Brazil than men [26]. Additionally, men and older individuals suffer from more fatal chronic diseases [25,26].

Our study presents some limitations that should be highlighted. Statistical inference techniques were used when considering that the patients with CCC have similar living conditions because they were from rural areas of small municipalities and were living in unfavorable economic conditions. The modest explanation of the variability of previous BZN use observed by the models (pseudo R^2^) may have been a result of individual and contextual factors that were not included in the present study, such as geopolitical or local healthcare service features that may influence the prescription or use of BZN among CCC patients. In addition, the cross-sectional design of this study does not allow us to establish causal relationships. Finally, an important limitation is the fact that the information was obtained from the self-report of patients with CCC, and this approach is subject to measurement bias, either intentional or memory bias. Despite these limitations, we believe that the investigation of this scenario is important, useful and necessary because it clarifies the health conditions of patients who live outside of modern society. The investigation of the profile of previous BZN use among patients living in CD endemic areas can help reduce inequalities in the treatment of this disease and open doors for future investigations. Following these participants in a cohort study, which is currently underway, will provide us with more information on this topic.

## Conclusion

Our data suggest that CD is still associated with low socioeconomic profiles and that its treatment remains neglected in several Brazilian rural areas. We showed that only 27% of CCC patients who were residents in the endemic regions of Brazil had been specifically treated with BZN and that less than 20% of all CCC patients followed the treatment recommendations. The absence of a consensus on the benefits of BZN treatment among patients in the chronic phase of CD, in addition to a late diagnosis of CD and the difficulties of maintaining proper healthcare in poor rural areas with difficult access, appear to contribute significantly to this low adherence to BZN treatment.
